# Effect of antidepressants on neurodegeneration and neuroplasticity in patients with depression: A comparison between SSRI and SNRI

**DOI:** 10.1192/j.eurpsy.2025.796

**Published:** 2025-08-26

**Authors:** D. Sharma, S. C. Sarangi, S. Bhargava, B. N. Patra, R. Sagar, P. Pankaj, S. Kumaran

**Affiliations:** 1 Pharmacology; 2 Psychiatry; 3 Department of Nuclear Magnetic Resonance, All India Institute of Medical Sciences, New Delhi, India

## Abstract

**Introduction:**

Depression affects 57 million people in India and is often linked to neurodegeneration (10-90% of cases). However, there’s insufficient evidence comparing the effectiveness of SSRIs and SNRIs in managing neurodegeneration symptoms.

**Objectives:**

This prospective observational study aims to compare the effects of SSRI and SNRI monotherapy on neurodegeneration, neuroplasticity, and social cognition.

**Methods:**

This prospective observational study aims to compare the effects of SSRI and SNRI monotherapy on neurodegeneration, neuroplasticity, and social cognition.

Treatment-naïve patients with unipolar depression were evaluated for treatment response using the Hamilton Depression Rating Scale (HDRS) and neurodegeneration parameters at enrollment and after six weeks of antidepressant treatment. Neurodegenerative serum biomarkers [indoleamine-2,3-dioxygenase (IDO), neurofilament light chain protein (NLCP), brain derived neurotrophic factor (BDNF)] were assessed using ELISA. Social cognition was assessed using Social Cognition Rating tools in Indian setting (SOCRATIS). Neuroplasticity was assessed by resting state MRI.

**Results:**

A total of 150 patients of unipolar depression were enrolled, out of these n=126 patients were prescribed SSRI and 24 patients were prescribed SNRI. Both SSRI and SNRI group have significant reduction in HDRS score at 6-week compared to baseline (both p<0.001), but no intergroup difference. Overall treatment responder rate (HDRS score reduction >50%) was 11.33%, but SSRI group has more responder (12.69%) compared to SNRI (4.16%). After 6 weeks of follow-up, serum IDO in SSRI group and NLCP levels in both groups were significantly decreased when compared to baseline (p<0.001) and BDNF levels were significantly increased in SSRI group when compared to baseline (p<0.01). As per SOCRATIS, after 6 weeks treatment, SSRI and SNRI didn’t show any significant difference. fMRI assessment of depression patients showed significant decrease in cortical thickness of inferiortemporal, parsopercularis and precuneus regions of brain (p<0.05) in comparison with healthy controls. But there was no significant difference/increase in cortical thickness after 6 weeks of follow-up when compared to baseline.

**Conclusions:**

After six weeks of antidepressant treatment, the treatment responder rate among all depression patients was 11.33%, with better outcomes observed in the SSRI group compared to the SNRI group. Likewise, in the assessment of social cognition and neurodegeneration-related biomarkers, the SSRI group showed superior performance over the SNRI group.

**Disclosure of Interest:**

None Declared

